# 端粒酶与CYFRA21-1联合测定对肺癌所致恶性胸腔积液同良性胸腔积液鉴别的诊断意义

**DOI:** 10.3779/j.issn.1009-3419.2010.06.017

**Published:** 2010-06-20

**Authors:** 红梅 李, 军桦 付, 元德 修, 清华 周

**Affiliations:** 1 300052 天津，天津医科大学总医院肺癌研究所 Lung Cancer Institute, Tianjin Medical University General Hospital, Tianjin 300052, China; 2 266003 青岛，青岛大学医学院附属医院肿瘤中心 Cancer Center, the Affiliated Hospital of Medical College Qingdao University, Qingdao 266003, China

**Keywords:** 端粒酶, CYFRA21-1, 胸腔积液, 鉴别诊断, Telomerase, CYFRA21-1, Pleural effusion, Differential diagnosis

## Abstract

**背景与目的:**

恶性胸腔积液中端粒酶和CYFRA21-1均可呈阳性表达，单个肿瘤标志物测定存在敏感性和特异性均较低的问题。本文旨在探讨端粒酶和CYFRA21-1联合测定对肺癌所致恶性胸腔积液同良性胸腔积液的鉴别诊断意义。

**方法:**

80例肺癌致恶性胸腔积液和50例良性胸腔积液患者，采用端粒重复序列扩增-酶联免疫吸附法检测胸腔积液端粒酶活性，用酶免疫分析法检测CYFRA21-1水平，对测定结果进行统计学处理。

**结果:**

恶性胸腔积液端粒酶和CYFRA21-1水平明显高于良性胸腔积液，差异有统计学意义（*t*=17.252和*t*=13.951，*P* < 0.001）。端粒酶活性诊断的敏感性为71.3%，特异性为86.0%，准确性为76.9%。CYFRA21-1诊断的敏感性为60.0%，特异性为78.0%，准确性为66.9%。两者联合测定的敏感性为90.0%，特异性为76.0%，准确性为86.9%。联合测定的敏感性和准确性均高于单项测定（*χ*^2^=9.002和*χ*^2^=19.201，*P* < 0.01；*χ*^2^=4.389和*χ*^2^=14.647，*P* < 0.05）。

**结论:**

端粒酶和CYFRA21-1联合测定可提高良恶性胸腔积液鉴别诊断的敏感性和准确性。

## Abstract (en)

**Background and objective:**

Telomerase and CYFRA21-1 may be positively expressed in malignant pleural effusion, but the sensitivity and specificity of single tumor marker were low. The aim of this study is to investigate the diagnostic value of combining determination of telomerase activity and CYFRA21-1 levels in differentiating benign from malignant pleural effusion caused by lung cancer.

**Methods:**

80 patients with malignant and 50 patients with benign pleural effusion were enrolled into this study. The telomerase activity in pleural effusion was tested by means of telomeric repeat amplification protocal-PCR-ELISA (TRAP-PCR-ELISA) and CYFRA21-1 levels were tested by the EIA method. All the results were analyzed by the statistical method.

**Results:**

The levels of telomerase and CYFRA21-1 in malignant pleural effusion was significantly higher than that in benign one (*t*=17.252 and *t*=13.951, *P* < 0.001). The sensitivity of telomerase activity testing for diagnosing malignant pleural effusion was 71.3%; the specificity was 86.0% and the overall accuracy was 76.9%. The sensitivity of CYFRA 21-1 testing was 60.0%, the specificity was 78.0% and the overall accuracy was 66.9%. The sensitivity of the combined testing was 90.0%, the specificity was 76.0% and the overall accuracy was 86.9%. The sensitivity and the overall accuracy of combined testing were higher than those of telomerase and CYFRA21-1 testing single(*χ*^2^=9.002 and *χ*^2^=19.201, *P* < 0.01; *χ*^2^=4.389 and *χ*^2^=14.647, *P* < 0.05).

**Conclusion:**

The combined testing of telomerase with CYFRA21-1 can increase the sensitivity and overall accuracy of differential diagnosis of benign and malignant pleural effusion diagnosis.

胸腔积液可由多种良恶性疾病引起，性质不同，治疗方案及预后均不同。目前，常用于胸腔积液鉴别诊断的有胸水常规、生化、细胞学及癌胚抗原等方法，但其灵敏性、特异性及准确性均较低。胸膜活检和胸腔镜等检查的准确性高，但创伤大、价格高。临床上仍有20%-30%的患者在使用常规方法仍不能明确胸腔积液的性质。近年研究^[1, 2]^表明，端粒酶和细胞角蛋白19片段（CYFRA 21-1）在恶性胸腔积液中呈阳性表达，可用于恶性胸腔积液的鉴别诊断。

本文采用端粒重复序列扩增-酶联免疫吸附实验法（TRAP-PCR-ELISA）和酶免疫分析法（EIA）分别测定了80例恶性胸腔积液和50例良性胸腔积液患者胸水中端粒酶活性和CYFRA21-1水平，探讨联合检测此两项肿瘤标志物在良恶性胸腔积液中的鉴别诊断价值，报道如下。

## 1 材料与方法

### 1.1 病例选择

选择青岛大学医学院附属医院2007年1月-2008年12月收治的130例胸腔积液患者，其中肺癌伴胸腔积液患者80例，男50例，女30例，年龄49岁-76岁，平均年龄59.5岁。均有确切的病理组织学或细胞学诊断依据，其中鳞癌39例，腺癌21例，小细胞癌15例，大细胞癌5例。良性胸腔积液患者50例，男32例，女18例，年龄47岁-73岁，平均年龄57.5岁，患者均依据临床表现、结核菌素试验、胸水常规生化、细菌培养等检查确诊，其中结核性胸膜炎25例，肺炎18例，支气管扩张5例，肺脓肿2例。

### 1.2 端粒酶活性测定

常规行胸腔穿刺术抽取新鲜胸水100 mL送检，离心（4 ℃, 1 200 r/min, 10 min），弃上清，离心细胞置液氮中保存（-196 ℃）。检测前用预冷的PBS缓冲液（pH 7.4）漂洗细胞2次，分别离心（4 ℃, 1 200 r/min, 10 min），弃上清。加预冷的端粒酶裂解缓冲液，0 ℃孵化30 min，离心（4 ℃, 12 000 r/min, 20 min），吸出上清液，取少许测定蛋白浓度，将细胞提取物稀释至3 μg/μL。采用TRAP-ELISA法，将上述各种标本的沉淀细胞（约10^5^-10^6^）置于1.5 mL离心管，150 μL裂解液抽提端粒酶。端粒酶活性和聚合酶链反应（PCR）扩增反应系统：5×端粒重复扩增反应混合液，10 μL；TaqDNA多聚酶（5 U/μL），0.4 μL；再加2 μL样品提取液；37.6 μL蒸馏水。采用PCR扩增仪，酶反应：30 ℃、30 min。PCR循环扩增：94 ℃、30 s，55 ℃、30 s，35个循环。阴性对照加2 μL裂解液；阳性对照加1 μL端粒酶质控模板溶液。扩增产物经包被好的微量96孔板捕获后，加人辣根过氧化物酶标记的抗二硝基苯抗体（Anti-DNP）孵育1 h后洗涤，四甲基联苯胺（TMB）显色10 min。MK3酶标仪（Dragon Lab System公司）上用双波长法测定各孔波长450 nm吸光度（*A*450）、波长690 nm吸光度（*A*690）的值，*A*690为参考波长，用于消除孔间误差。端粒酶活性为△*A*=*A*450-*A*690。△*A*大于阴性对照孔的2.0倍即判定为端粒酶阳性。敏感性=恶性胸水患者中阳性例数/恶性胸水患者总数；特异性=良性胸水患者中阴性例数/良性胸水患者总数；准确性=（恶性胸水患者中阳性例数+良性胸水患者中阴性例数）/患者总数。

### 1.3 CYFRA21-1测定

取胸腔积液5 mL采用酶免疫分析法（EIA）测定CYFRA21-1。试剂盒由德国Boehringer Mannheim公司提供。阳性判断标准为CYFRA21-1 > 10 ng/mL，严格按试剂盒说明书要求操作。

### 1.4 统计学处理

采用SPSS 12.0统计软件进行统计学分析，计算敏感性、特异性和准确性，进行综合评价。计量资料以Mean±SD表示，样本均数采用*t*检验，计数资料采用*χ*^2^检验，*P* < 0.05为差异有统计学意义。

## 2 结果

### 2.1 良恶性胸腔积液端粒酶活性和CYFRA21-1水平检测结果

在80例肺癌致恶性胸腔积液中，有35例发现了恶性肿瘤细胞，端粒酶活性为0.395±0.157，其余45例未找到恶性肿瘤细胞，端粒酶活性为0.347±0.108。良恶性胸腔积液端粒酶活性和CYFRA21-1水平检测结果见表 1，恶性胸腔积液组端粒酶活性和CYFRA21-1水平均明显高于良性胸腔积液组，差异有统计学意义（*P* < 0.001）。

**1:** 良恶性胸腔积液端粒酶活性和CYFRA21-1水平的表达（Mean±SD） The expression of telomerase activity and CYFRA21-1 level in benign and malignant pleural effusion (Mean±SD)

Tumor marker	Benign pleural effusion (*n*=50)	malignant pleural effusion (*n*=80)	*t*	*P*
Telomerase (A value)	0.075±0.009	0.376±0.123	17.252	0.001
CYFRA21-1 (nq/mL)	2.090±1.420	18.930±8.450	13.951	0.001

### 2.2 端粒酶和CYFRA21-1单项及联合检测对恶性胸腔积液的诊断价值

由表 2可见，两项肿瘤标记物联合检测若以一项阳性作为恶性胸腔积液的诊断标准，则联合检测敏感性为90.0%，明显高于端粒酶和CYFRA21-1单项检测的敏感性71.3%和60.0%（*χ*^2^=9.002和*χ*^2^=19.201，*P* < 0.01和*P* < 0.001），特异性虽下降为76%，但准确性升高为86.9%，准确性差异亦有统计学意义（*χ*^2^=4.389和*χ*^2^=14.647，*P* < 0.05和*P* < 0.001）。

**2:** 端粒酶和CYFRA21-1检测对恶性胸腔积液的诊断价值 The diagnotic value of telomerase and CYFRA21-1 in malignant pleural effusion

Diagnotic value	Specificity (%)	Overall accuracy (%)	*χ*^2^	P	Sensitivity (%)	*χ*^2^	P
Telomerase 1	86.0 (43/50)	76.9(100/130)^#^	4.389	< 0.05	71.3(57/80)^*^	9.002	< 0.01
CYFRA21-12	78.0 (39/50)	66.9 (87/130)^Δ^	14.647	< 0.001	60.0 (48/80)^Δ^	19.201	< 0.001
Combinations	82.0 (41/50)	86.9(113/130)			90.0 (72/80)		
Sensitivity: 3 *vs* 1, *χ*^2^=9.002, ^*^: *P* < 0.01; 3 *vs* 2, *χ*^2^=19.201, ^Δ^: *P* < 0.001. Overall accuracy: 3 *vs* 1, *χ*^2^=4.389, ^#^: *P* < 0.05; 3 *vs* 2, *χ*^2^=14.647, ^Δ^: *P* < 0.001.							

## 3 讨论

端粒是真核生物染色体末端包含着独特重复片段的特殊结构，它能催化染色体末端的特殊结构端粒复制，它存在于人的生殖细胞、永生化细胞系、肿瘤细胞和增殖组织。恶性肿瘤细胞的一个重要生物学特征是永生化，这与端粒酶激活有关。且端粒酶活性的表达与肿瘤的发生、发展都有相关性^[3, 4]^。研究^[5]^表明，大多数肿瘤组织细胞均有较高水平的端粒酶表达，而在正常组织和体细胞为阴性，因此可作为癌症筛选的标记物。肿瘤的胸膜转移和胸膜原发肿瘤等引起的胸腔积液与炎性胸腔积液，其端粒酶活性的差异可用于鉴别良恶性胸腔积液。文献^[6]^报道肺癌组织中端粒酶阳性率达80%，恶性胸液中端粒酶阳性率约为47.5%-91.4%。

CYFRA21-l是细胞角蛋白19片段，由多种上皮细胞表达，是一种分子量为40 ku-68 ku的细胞结构蛋白，主要分布于肺组织尤其是肺肿瘤上皮细胞浆中，可在激活的蛋白酶作用下被降解或在细胞死亡后以溶解片段形式释放入血清中^[7]^。研究^[8]^表明：恶性胸腔积液患者血和胸水中CYFRA21-1水平均明显增高，且以胸水中升高明显，其对恶性胸腔积液诊断敏感性约50%-77%，特异性约60%-79%。

本研究选择80例恶性胸腔积液患者和50例良性胸腔积液患者，分别检测其胸水中端粒酶活性和CYFRA21-1水平，结果发现恶性胸腔积液患者端粒酶活性明显高于良性胸腔积液患者，CYFRA21-1水平亦明显高于良性胸腔积液患者，与相关文献报道一致^[9, 10]^。故端粒酶和CYFRA21-1均可作为良恶性胸腔积液鉴别诊断的辅助手段。

由于肿瘤细胞具有异质性，迄今为止尚未发现对各型癌症均具有高度特异性和敏感性的标志。众多研究者已达成共识，通过采用联合检测可提高诊断效能，弥补单项标志对不同组织类型肿瘤敏感性不同的缺点。本实验结果也显示，端粒酶和CYFRA21-1两项肿瘤标志联合测定若以一项阳性作为恶性胸腔积液的诊断标准，则敏感性为90.0%（72/80），特异性为76.0%（38/50），准确性为86.9%（113/130）；若以两项均阳性作为诊断标准，敏感性下降至56.3%（45/80），特异性可达94.0%（47/50），准确性下降至70.8%（92/130）。因此，临床如有一项阳性，要考虑恶性胸腔积液的诊断，并作进一步检查；如两项同时阳性则诊断为恶性胸腔积液的特异性高。上述结果表明联合测定胸腔积液中端粒酶和CYFRA21-1水平，可提高恶性胸腔积液的诊断率，尤其对良性和恶性胸腔积液的鉴别诊断具有重要的意义。
